# Exogenous Fe^2+^ alleviated the toxicity of CuO nanoparticles on *Pseudomonas tolaasii* Y-11 under different nitrogen sources

**DOI:** 10.7717/peerj.10351

**Published:** 2020-11-10

**Authors:** Yuran Yang, Can Zhang, Xuejiao Huang, Xuwei Gui, Yifang Luo, Zhenlun Li

**Affiliations:** 1 Chongqing Key Laboratory of Soil Multi-Scale Interfacial Process, College of Resources and Environment, Southwest University, Chongqing, China; 2 Chongqing Key Laboratory of Plant Disease Biology, College of Plant Protection, Southwest University, Chongqing, China

**Keywords:** Ferrous ion, CuO-NPs, Nitrogen removal, Detoxify, Functional microorganisms

## Abstract

Extensive use of CuO nanoparticles (CuO-NPs ) inevitably leads to their accumulation in wastewater and toxicity to microorganisms that effectively treat nitrogen pollution. Due to the effects of different mediums, the sources of CuO-NPs-induced toxicity to microorganisms and methods to mitigating the toxicity are still unclear. In this study, CuO-NPs were found to impact the nitrate reduction of *Pseudomonas tolaasii* Y-11 mainly through the action of NPs themselves while inhibiting the ammonium transformation of strain Y-11 through releasing Cu^2+^. As the content of CuO-NPs increased from 0 to 20 mg/L, the removal efficiency of NO_3_^−^ and NH_4_^+^ decreased from 42.29% and 29.83% to 2.05% and 2.33%, respectively. Exogenous Fe^2+^ significantly promoted the aggregation of CuO-NPs, reduced the possibility of contact with bacteria, and slowed down the damage of CuO-NPs to strain Y-11. When 0.01 mol/L Fe^2+^ was added to 0, 1, 5, 10 and 20 mg/L CuO-NPs treatment, the removal efficiencies of NO_3_^-^ were 69.77%, 88.93%, 80.51%, 36.17% and 2.47%, respectively; the removal efficiencies of NH_4_^+^ were 55.95%, 96.71%, 38.11%, 20.71% and 7.43%, respectively. This study provides a method for mitigating the toxicity of CuO-NPs on functional microorganisms.

## Introduction

The excessive use of nitrogen fertilizer coupled with the indiscriminate discharge of domestic sewage and industrial wastewater has caused increasingly severe nitrogen pollution (Liu et al., 2020; Paredes et al., 2020). Nitrogen pollution causes serious harm to the ecological environment, such as eutrophication of water, and causes health problems such as infant methemoglobinemia (Liu et al., 2020; Paredes et al., 2020; Zhang et al., 2020a; Zhang et al., 2016). Microbial nitrogen removal is an economical and effective method controlling nitrogen pollution in water. Biological nitrification and denitrification are therefore widely used for pollution control (He et al., 2020). However, the functional activities of those microorganisms are highly susceptible to external influences, such as nanoparticles (NPs) (Wang et al., 2017a; Wang et al., 2017b; Zhang et al., 2018a).

In recent years, with the rapid development of nanotechnology, NPs have been widely used in various consumer and industrial products (Nowack et al., 2015; Zhang et al., 2018a). For example, CuO-NPs are widely used in gas sensors, antibacterial textiles, batteries, catalysts, and metal coatings (Chen et al., 2019; Wang et al., 2017a). The increasingly widespread application of CuO-NPs inevitably leads to the release of CuO-NPs into industrial and municipal wastewater and accumulation in activated sludge (Hou et al., 2015; Jośko et al., 2019; Wang et al., 2017a; Zhang et al., 2018a; Zhang et al., 2017b). It was speculated that the concentration of CuO-NPs was about 1 mg/L in the environment and 50 mg/L in semiconductor wastewater (Zhang et al., 2020b; Zhang et al., 2018a). The introduction of NPs into the sewage treatment system will seriously threaten the activity of sewage treatment microorganisms, thus causing damage to the sewage treatment system. CuO-NPs are toxic to microorganisms in activated sludge. They have been found to destroy the integrity of the microbial cell membrane in activated sludge, reduce the diversity and activity of the bacterial community, and significantly reduce the effectiveness of nitrogen and phosphorus removal (Wang et al., 2017a; Wang et al., 2017b; Zhang et al., 2018a). Hou et al. (2016) found that the denitrification process of the biofilm reactor was inhibited by 50 mg/L CuO-NPs, reducing the nitrogen removal efficiency and significantly reducing the activity of nitrite reductase (NIR) and nitrate reductase (NAR). Huang et al. (2020) found that the propagation and ammonium removal of *Pseudomonas putida* strain Y-9 were inhibited significantly by 1 mg/L CuO-NPs. Wang et al. (2017b) found that 1 mg/L CuO-NPs had a significant inhibitory effect on ammonia monooxygenase (AMO) and nitrite oxidoreductase (NOR) activity.

There is no unified answer as to whether the toxicity of NPs is caused by the NPs themselves or the released metal ions (Huang et al., 2020; Wang et al., 2016). It is generally believed that metal oxide NPs can dissolve in aqueous media, causing heavy metal ions to be released into the media (Luo et al., 2018; Wang et al., 2016). Therefore, improving the stability of NPs in aqueous solutions can mitigate their toxicity to microorganisms. Studies have shown that the addition of organic matter can alleviate the damage caused by NPs to bacteria. Zhao et al. (2013) showed that the addition of fulvic acid could reduce the damage to the bacterial membrane caused by CuO-NPs. However, studies have also shown that when the organic matter content in the wastewater was high, the chemical oxygen demand (COD) content in the water body was increased, which was not conducive to the removal of nitrogen and the growth of bacteria (Pan et al., 2020). Therefore, other methods of mitigating the impact of NPs on functional bacteria need to be sought.

Some scholars improved the matrix of NPs by doping with iron to reduce the cytotoxicity of NPs. For example, Adeleye et al. (2018) and Naatz et al. (2017) reduced the toxicity to marine phytoplankton and zebrafish by doping Fe in CuO-NPs. The solubility and toxicity of ZnO NPs were significantly reduced by doping Fe in ZnO NPs (Fairbairn et al., 2011; George et al., 2010; Xia et al., 2011). However, the method of changing the CuO-NPs matrix to relieve cytotoxicity is not suitable for sewage treatment plants. Fe^2+^ is an essential element for microbial growth (Feng et al., 2020). The use of iron in sewage treatment to enhance nitrogen removal has been extensively studied (Feng et al., 2020; Liu et al., 2020; Liu & Ni, 2015; Qiao et al., 2013). However, there has been no research on the direct addition of Fe^2+^ in wastewater treatment to reduce the toxicity of NPs to functional bacteria.

In view of the fact that there are few studies on the differences in CuO-NP cytotoxicity given different nitrogen sources, this study used *Pseudomonas tolaasii* Y-11 which exhibits excellent denitrification and nitrification activity, as the bacterial source (He et al., 2016). A series of experiments were conducted to: (1) determine the effect of CuO-NPs on the proliferation and nitrogen removal of strain Y-11; (2) reveal the effect of CuO-NPs on ammonium and nitrate removal performance of strain Y-11; and (3) evaluate the detoxification ability of Fe^2+^ on CuO-NPs. We hoped to provide a method for mitigating the toxicity of CuO-NPs on functional microorganisms.

## Materials & Methods

### Cell cultivation and culture media

The aerobic denitrification and heterotrophic nitrification strain *Pseudomonas tolaasii* Y-11 (KP410741) used in this study was isolated from a winter paddy field by He & Li (2015).

The basal medium (BM) (Huang et al., 2020) was used to determine the nitrogen removal performance of strain Y-11 (1 L containing 0.31 g NaNO_3_ (BM_1_) or 0.25 g (NH_4_)_2_SO_4_ (BM_2_), 2.56 g CH_3_COONa, 1.5 g KH_2_PO_4_, 0.42 g Na_2_HPO_4_, and 0.1 g MgSO_4_ ⋅ 7H_2_O). BM_1_ and BM_2_were the BM for two different nitrogen sources. We added 0.05 g/L FeSO_4_ ⋅ 7H_2_O to the BM to explore the mitigation effect of Fe^2+^ on CuO-NP toxicity. Before exposure to CuO-NPs, strain Y-11 was cultivated in Luria-Bertani (LB) medium (1 L containing NaCl 10 g, tryptone 10 g, and yeast extract 5 g) at 15 °C and 150 rpm/min for 36 h.

The initial pH of all the media was adjusted to 7.3 with 0.1 M NaOH or 0.1 M HCl. Each 250 ml conical flask contained 100 ml of medium. The medium was sterilized at 121 °C, 0.11 MPa for 20 min and cooled naturally to room temperature.

### Preparation of CuO-NPs suspension

The CuO-NPs (40 nm, 99.5% purity) used in this study were purchased from Zewu Company (Chongqing, China) as a dry powder. The suspension of the CuO-NPs (2000 mg/L) was prepared by adding 100 mg of the CuO-NPs to 50 mL ultrapure deionized water. Subsequently, the CuO-NPs suspension was sonicated (600 W and 40 kHz) for 20 min to increase their dispersion (Huang et al., 2020). The morphology of CuO-NPs was observed by a scanning electron microscope (SEM, Phenom World, Holland). The hydrodynamic diameter of ZnO-NPs in the suspensions were measured by Dynamic Light Scattering (DLS, Brookhaven, USA). A SEM image and hydrodynamic diameter of the CuO-NPs (2,000 mg/L) used for this study are available in the Figs. S1 and S2. Different concentrations of CuO-NPs in simulated sewage were obtained by diluting the suspension with basal medium.

### *P. tolaasii* Y-11 exposure to CuO-NPs with or without Fe

To investigate the difference in toxicity of CuO-NPs to strain Y-11 with different nitrogen sources, the strain was exposed to 1, 5, 10, and 20 mg/L CuO-NPs in the basal medium. Basal medium without CuO-NPs was used as a control. Strain Y-11 was inoculated into BM, and shaken for 48 h at 15 °C and 150 rpm. The inoculation volume of the above experiments was 1% (v/v), and the initial optical density (OD_600_) was about 0.1. The above experiments were repeated three times. After 48 h, the OD_600_ of the medium was measured. The medium was centrifuged (8000 rpm, 5 min) to determine the amount of NO_3_^−^, NH_4_^+^, and metal ions (Cu^2+^). Three parallel measurements were preformed per sample.

### *P. tolaasii* Y-11 exposure to Cu^2+^ with or without Fe^2+^

To investigate whether the toxicity of CuO-NPs was caused by dissolved Cu^2+^, the concentrations of Cu^2+^ in BM_1_ and BM_2_ were set at 0.01, 0.1, 0.5, and 1 mg/L and 0.01, 0.05, 0.1, and 0.15 mg/L, respectively. The above experiments were repeated in triplicate. The culture conditions and measurement indexes were the same as above.

### Analytical methods

The OD_600_ was measured at an optical density of 600 nm. NO_3_^−^ and NH_4_^+^ were detected in the supernatant after centrifugation (8000 rpm, 5 min) according to the method of He et al. (2019). Specifically, the concentrations of NO_3_^−^ and NH_4_^+^ were determined by the hydrochloric acid photometric method and the indophenol blue method, respectively. The concentration of Cu^2+^ in the supernatant was analyzed via ICP-OES (ICP-OES 5110, Agilent, USA).

The removal efficiency of NO_3_^−^ or NH_4_^+^ was calculated as follows:

(1) \begin{eqnarray*}R=({T}_{0}-{T}_{1})/{T}_{0}\times 100.0\text{%},\end{eqnarray*}

where *R* is the NO_3_^−^ or NH_4_^+^ removal efficiency (%), and *T*_0_ and *T*_1_ represent the initial and final NO_3_^−^ or NH_4_^+^ concentrations in the system, respectively.

### Statistical analyses

The significance of the results was tested using the one-way analysis of variance (ANOVA) in SPSS Statistics 20. Three parallel measurements were performed per sample and the results were expressed as mean ± standard deviation.

## Results and Discussion

### Effects of CuO-NPs on proliferation and nitrogen removal of strain Y-11 with and without Fe^2+^ addition

**Figure 1:**
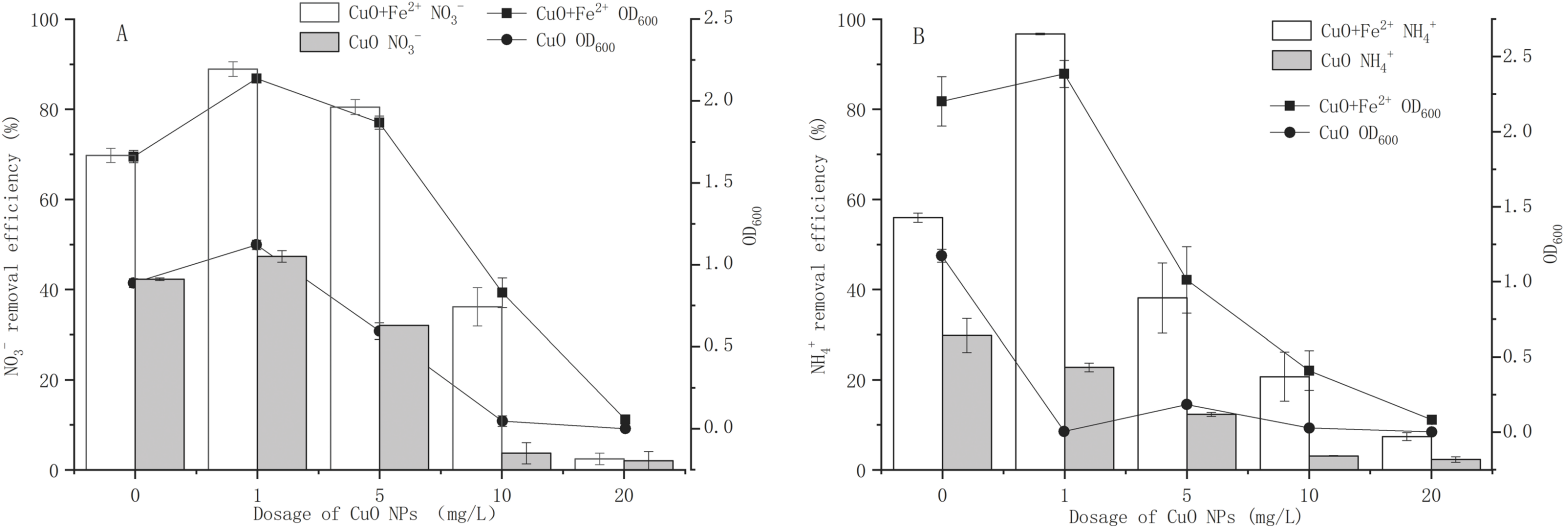
Effects of CuO NPs on growth and nitrogen removal of strain Y-11 with and without Fe^2+^ addition. (A) The effect of CuO NPs on the growth and NO${}_{3}^{-}$ removal of strain Y-11 in NO${}_{3}^{-}$ medium; (B) the effect of CuO NPs on the growth and NH${}_{4}^{+}$ removal of strain Y-11 in NH${}_{4}^{+}$ medium.

The effect of CuO-NPs on the proliferation and nitrogen removal performance of strain Y-11 with or without added Fe^2+^ is shown in Fig. 1. Interestingly, 1 mg/L CuO-NPs promoted cell proliferation and NO_3_^−^ removal (Fig. 1A). In the Fe ^2+^-free treatment, as the CuO-NP content increased, cell proliferation was inhibited. At 20 mg/L CuO-NPs, the proliferation of bacteria stopped completely. Meanwhile, the NO_3_^−^ removal efficiency also decreased from 42.29% to 2.05%. Hou et al. (2015) found that 50 mg/L CuO-NPs inhibited the denitrification process and significantly reduced the nitrogen removal rate. The addition of Fe^2+^ significantly promoted the growth of bacteria and the removal of NO_3_^−^. At 0, 1, 5, and 10 mg/L CuO-NPs, the OD_600_ reached 1.33, 2.14, 1.87, and 0.83, which were significantly higher values than those of Fe^2+^-free treatment. At 20 mg/L CuO-NPs, the bacteria grew slowly. In the Fe^2+^-containing treatment, when CuO-NP content was 0, 1, 5, 10, and 20 mg/L, the removal efficiencies of NO_3_^−^ were 69.77%, 88.93%, 80.51%, 36.17%, and 2.47%, respectively. The addition of Fe^2+^ increased the activity of the bacteria and enhanced the resistance to CuO-NPs. Song et al. (2016) found that exogenous Fe^2+^ could improve the microbial activity of water samples and promote the removal of NO_3_^−^. Therefore, adding Fe^2+^ is a feasible method by which to increase bacterial tolerance to CuO-NPs.

In the Fe^2+^-free treatment, as the CuO-NPs content increased from 0 to 20 mg/L, OD_600_ decreased from 1.17 to 0, and the NH_4_^+^ removal efficiency decreased from 29.83% to 2.33% (Fig. 1B). Even 1 mg/L CuO-NPs were inhibitory to bacteria. This was consistent with the research results of Huang et al. (2020). Zhang et al. (2017a) showed through their research that 1 mg/L CuO-NPs slightly affected the removal of ammonia, and 3–10 mg/L promoted the removal of ammonia. This was different from the results of the present study. By comparing the cell proliferation under Fe^2+^-free treatment, it could be concluded that the cytotoxicity of CuO-NPs in NH_4_^+^ wastewater was stronger than that of NO_3_^−^ wastewater. The cytotoxicity levels of CuO-NPs in BM_1_ and BM_2_ were different, and nitrification-related enzymes were more sensitive to CuO-NPs than denitrification-related enzymes. Ye et al. (2020) found that the denitrification process was more sensitive to the toxicity of ZnO NPs than the nitrification process. This finding was consistent with the results of this study. In the Fe^2+^-containing treatment, when the CuO-NP contents were 0, 1, 5, 10, and 20 mg/L, OD_600_ was 2.20, 2.39, 1.01, 0.41 and 0.08, respectively, and the NH_4_^+^ removal efficiencies were 55.95%, 96.71%, 38.11%, 20.71%, and 7.43%, respectively. It is worth noting that in the Fe^2+^-containing treatment, 1 mg/L CuO-NPs significantly promoted cell proliferation and NH_4_^+^ removal (compared to the control treatment). Fe plays an important role in the growth and metabolism of microorganisms and is a component of ferritin (Feng et al., 2020). Zhang et al. (2018b) found that Fe^2+^ (less than 5 mg/L) could significantly promote the removal of nitrogen in the ammonia oxidation system. Exogenous Fe^2+^ enhanced the resistance of strain Y-11 to CuO-NPs and promoted the removal of NH_4_^+^.

### Effect of Fe^2+^ addition on Cu^2+^ dissolution from CuO-NPs

**Figure 2:**
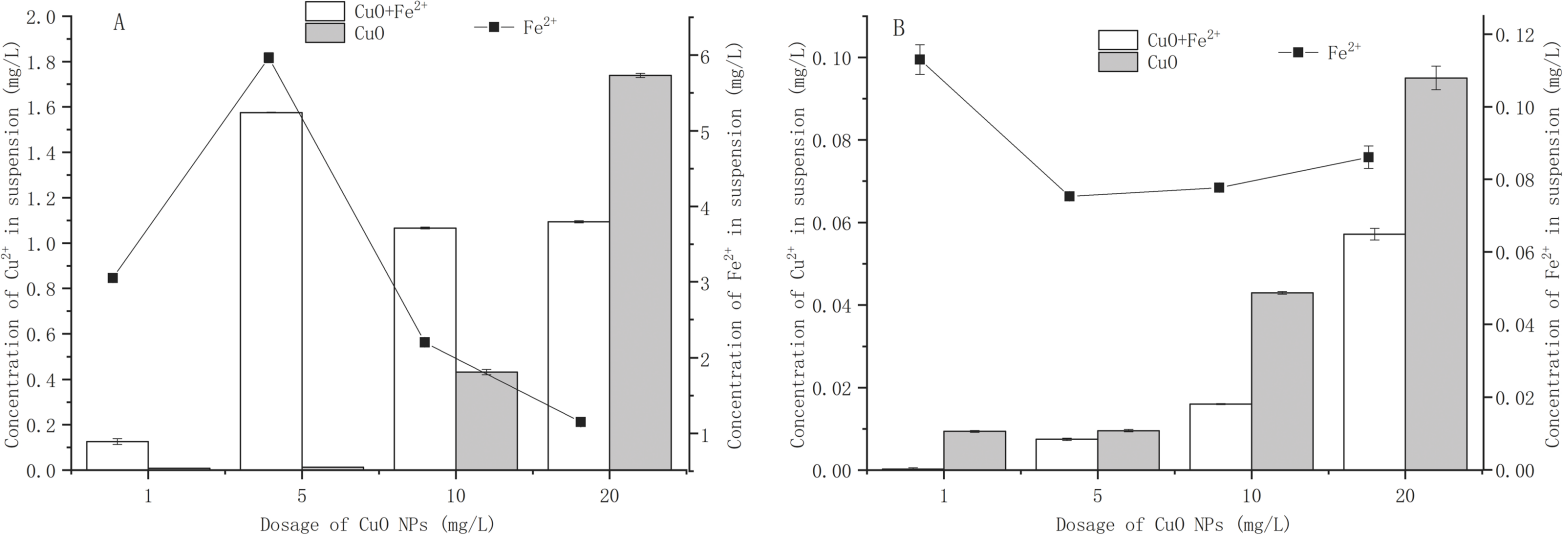
Effect of Fe^2+^ addition on Cu^2+^ dissolution from CuO NPs. (A) The effect of Fe^2+^ addition on Cu^2+^ dissolution from CuO NPs in NO${}_{3}^{-}$ medium; (B) the effect of Fe^2+^ addition on Cu^2+^ dissolution from CuO NPs in NH${}_{4}^{+}$ medium.

Due to the large specific surface area of CuO-NPs, Cu^2+^ would be released in the aqueous environment (Luo et al., 2018). Figure 2 shows the effect of Fe^2+^ addition on the release of Cu^2+^ from CuO-NPs. In the Fe^2+^-free treatment, the amounts of Cu^2+^ released by 1, 5, 10, and 20 mg/L CuO-NPs in BM_1_ were 0.008, 0.013, 0.432, and 1.739 mg/L, respectively (Fig. 2A). In the Fe^2+^- containing treatment, as the CuO-NPs content increased from 1 mg/L to 20 mg/L, the maximum release amount of Cu^2+^ of 1.575 mg/L occurred at 5 mg/L CuO-NPs. At 1 mg/L, 10 mg/L, and 20 mg/L CuO-NPs, the Cu^2+^ concentrations were 0.126 mg/L, 1.07 mg/L, and 1.09 mg/L, respectively. As the dose of NPs increased, the dissolved metal ions were adsorbed by the NPs, resulting in a decrease in the amount of metal ions released (Wang et al., 2016). Interestingly, although the addition of Fe^2+^ promoted the release of Cu^2+^ from CuO-NPs (except for 20 mg/L CuO-NPs), bacterial activity and the removal of NO_3_^−^ were enhanced (Fig. 1A). Kang, Mauter & Elimelech (2009) found that higher concentrations of divalent cations (such as Mg and Ca) in wastewater may cause nano-metal oxide particles to accumulate more than in river water, thereby affecting their ecotoxicity. Adeleye et al. (2018) found that doping Fe in CuO-NPs significantly promoted the release of Cu^2+^, but this did not increase the toxicity of CuO-NPs to the marine phytoplankton. However, Naatz et al. (2017) found that the doping of Fe significantly reduced the dissolution of Cu^2+^ and reduced the toxicity of CuO-NPs to zebrafish embryos. Therefore, in BM_1_, the toxicity of CuO-NPs to strain Y-11 was caused by the NPs themselves. Exogenous Fe^2+^ enhanced the tolerance of strain Y-11 to CuO-NPs.

CuO-NPs released very little Cu^2+^ in BM_2_ (Fig. 2B). As the CuO-NPs content increased from 1 mg/L to 20 mg/L, the released Cu^2+^ content gradually increased. In the Fe^2+^-free treatment, the maximum release amount of Cu^2+^ reached 0.095 mg/L when the CuO-NPs content was 20 mg/L. This was much lower than the release amount of Cu^2+^ in BM_1_ at the same concentration (1.739 mg/L). The dissolution of NPs is affected by their surface area (Liu et al., 2018). Under the action of electrostatic attraction, NH_4_^+^ ions were adsorbed on the surface of CuO-NPs, which reduced the surface area of the CuO-NPs. In the Fe^2+^-containing treatment, less Cu^2+^ was released. The Cu^2+^ release amounts were 0.0003, 0.0075, 0.016, and 0.057 mg/L at 1, 5, 10, and 20 mg/L of CuO-NPs, respectively. Fe^2+^ played a role in inhibiting the dissolution of Cu^2+^ from CuO-NPs. Previous studies had shown that the composition of the solution (such as a high concentration of divalent cations) affected the dissolution of NPs (Chen et al., 2017; Kang, Mauter & Elimelech, 2009; Kunhikrishnan et al., 2015). Mao et al. (2020) found that the addition of metal cations promoted the aggregation of NPs. Exogenous Fe^2+^ increased the content of divalent cations in the medium and inhibited the dissolution of CuO-NPs. Although the solubility of CuO-NPs in BM_2_ was lower, CuO-NPs had a higher inhibitory effect on bacterial proliferation and NH_4_^+^ removal (Fig. 1B). Exogenous Fe^2+^ inhibited the dissolution of Cu^2+^, and the toxicity of CuO-NPs to strain Y-11 was reduced. Therefore, in BM_2_, the toxicity of CuO-NPs on the strain Y-11 was mainly caused by the release of metal ions.

### Effects of Cu^2+^ on growth and nitrogen removal of strain Y-11 with and without Fe^2+^ addition

**Figure 3:**
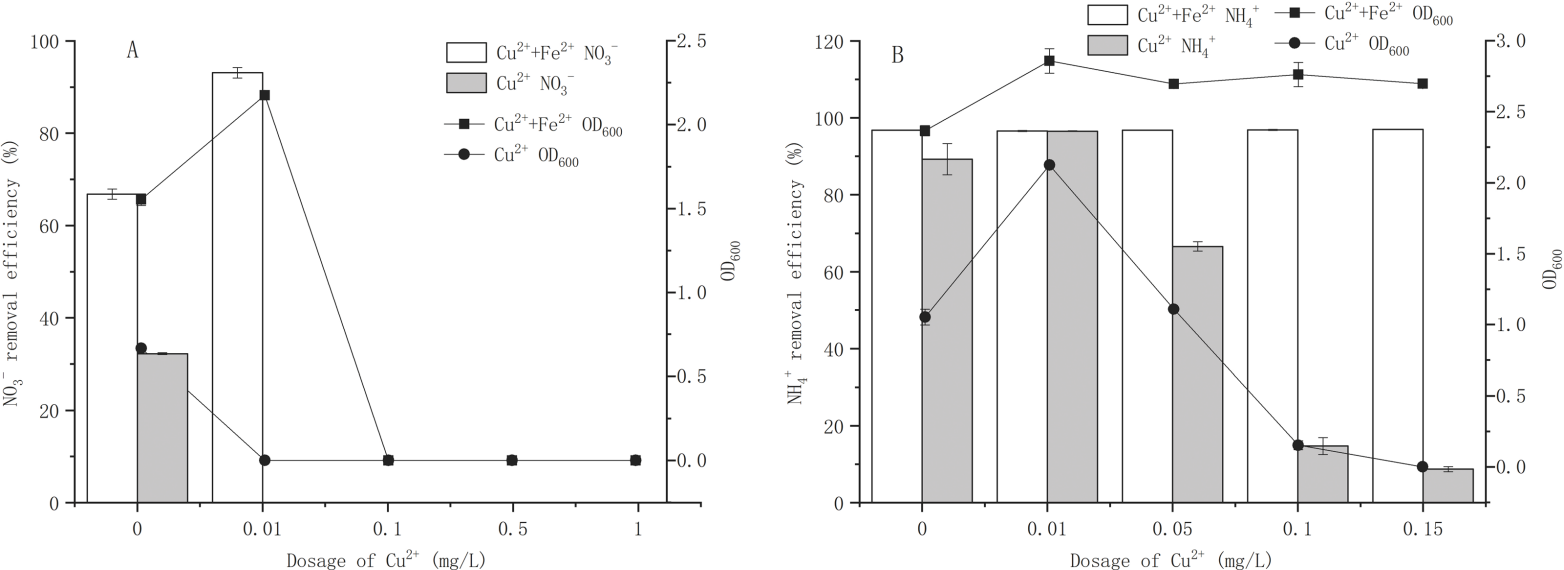
Effects of Cu^2+^ on growth and nitrogen removal of strain Y-11 with and without Fe^2+^ addition. (A) The effect of Cu^2+^ on the growth and NO ${}_{3}^{-}$ removal of strain Y-11 in NO${}_{3}^{-}$ medium; (B) the effect of Cu^2+^ on the growth and NH ${}_{4}^{+}$ removal of strain Y-11 in NH${}_{4}^{+}$ medium.

Metal ions are released from NPs in aqueous environment. According to the concentration of Cu^2+^ released from CuO-NPs in different nitrogen sources, the effects of Cu ^2+^ on cell proliferation and nitrogen removal of strain Y-11 were discussed (Fig. 3). In BM_1_, the release of Cu^2+^ exceeded 1 mg/L (Fig. 2A). Therefore, the concentrations of Cu^2+^ were set at 0, 0.01, 0.1, 0.5, and 1mg/L (Fig. 3A) for testing. Interestingly, when the concentration of Cu^2+^ exceeded 0.01 mg/L, severe toxicity was produced and cell proliferation stopped. At 0.01 mg/L Cu^2+^, the addition of Fe^2+^ improved the activity of bacteria, and the removal efficiency of NO_3_^−^ reached 93.1% (66.8% for the control treatment). Low-dose CuO-NPs (1 mg/L) released less than 0.1 mg/L Cu^2+^ (Fig. 2A), which may be the main reason for the low dose of CuO-NPs (<1 mg/L) induced the synthesis of related enzymes (such as Cu-containing nitrite reductase) and material transfer to increase the activity of the bacteria (Huang et al., 2020; Wang & Chen, 2016; Zhang et al., 2017a). The addition of Fe^2+^ promoted the release of Cu^2+^. When Fe^2+^ was added, the toxicity of CuO-NPs did not increase but promoted cell proliferation and NO_3_^−^ removal (Figs. 1A and 2A). However, the addition of Fe^2+^ in the treatment of Cu^2+^ did not achieve detoxification (Fig. 3A). Cu^2+^ had strong toxicity in cells. Therefore, we concluded that in BM_1_ the toxicity of CuO-NPs on strain Y-11 was mainly caused by the NPs themselves. The addition of exogenous Fe^2+^ caused the Fe^2+^ to be adsorbed onto the CuO-NPs and inhibited the direct contact between the CuO-NPs and cells, thereby reducing the damage caused by CuO-NPs to strain Y-11. Although exogenous Fe^2+^ promoted the release of Cu^2+^, Cu^2+^ may undergo a hydrolysis process to generate hydroxides and reduce the poisonous effects of Cu^2+^ (Wang et al., 2016).

In BM_2_, the maximum release amount of Cu^2+^ from CuO-NPs was less than 0.1 mg/L. We set the Cu^2+^ concentration gradient in BM_2_ to 0, 0.01, 0.05, 0.1, and 0.15 mg/L (Fig. 3B). In Fe^2+^-free wastewater, 0.01 mg/L Cu^2+^ significantly increased cell proliferation to 2.12 (1.05 for the Cu^2+^-free treatment). As the concentration of Cu^2+^ increased, cell proliferation was inhibited. The NH_4_^+^ removal efficiency decreased from 96.52% to 8.72% as Cu^2+^ concentration increased. The release of Cu^2+^ in BM_2_ was less, but the CuO-NPs showed a higher inhibition effect (Figs. 1B and 2B). With the addition of Fe^2+^, cell proliferation was maintained at about 2.5, and the NH_4_^+^ removal efficiency was higher than 96%. The addition of Fe^2+^ inhibited the release of Cu^2+^ from CuO-NPs and reduced its toxic effect on cells (Figs. 1B and 2B). In BM_2_, the aggregation of CuO-NPs due to the electrostatic effect effectively inhibited the distribution of CuO-NPs in the wastewater, and the free Cu^2+^ made a major contribution to the cytotoxicity. The addition of Fe^2+^ effectively reduced the dissolution of Cu^2+^ from CuO-NPs and reduced the toxic effects of Cu^2+^ on cells.

### Effect of exogenous Fe^2+^ on the aggregation behavior of CuO-NPs

The hydrodynamic diameter could reflect the aggregation state of CuO-NPs in water medium. The hydrodynamic diameter of CuO-NPs under different nitrogen sources was shown in Table 1. The hydrodynamic diameter increased gradually with the increase of the concentration of CuO-NPs, indicating that CuO-NPs had aggregated. This may be due to the increased collision frequency (Mwaanga, Carraway & Van den Hurk, 2014). The hydrodynamic diameter of CuO-NPs in BM_2_ is larger than that of in BM_1_, which further revealed that the amount of Cu^2+^ released in BM_2_ is less than that of in BM_1_. Furthermore, it has been reported that the aggregated NPs can reduce toxicity (Hou et al., 2016). Exogenous Fe^2+^ further promoted the aggregation of CuO-NPs, and the hydrodynamic diameter increases to 2–3 times of the original. The aggregation of CuO-NPs effectively reduced the possibility of contact with bacteria, and reduced the damage of CuO-NPs themselves to cells.

**Table 1:** Effect of Fe^2+^ on the hydrodynamic diameter of CuO-NPs.

Particle size (nm)		CuO-NPs concentration (mg/L)			
		1	5	10	20
NO_3_^−^ wastewater	Fe^2+^-free	–	659.95	743.73	1116.52
	Fe^2+^-containing	2,522.24	2,254.52	2,027.38	1,827.41
NH_4_^+^ wastewater	Fe^2+^-free	720.57	735.00	833.36	1,100.54
	Fe^2+^-containing	1,556.45	1,201.48	1,528.13	1,331.07

**Notes.**

– means not detected.

## Conclusions

The cytotoxicity of CuO-NPs in NO_3_^−^ medium and NH_4_^+^ medium was caused by the NPs themselves and the released Cu^2+^, respectively. CuO-NPs showed higher cytotoxicity in NH_4_^+^ medium than in NO_3_^−^ medium. Exogenous Fe^2+^ significantly promoted the aggregation of CuO-NPs, reduced the possibility of contact with bacteria, and slowed down the damage of CuO-NPs to strain Y-11. The results of this study provide a new method to alleviate the toxic effects of CuO-NPs on nitrogen-removing microorganisms.

##  Supplemental Information

10.7717/peerj.10351/supp-1 Supplemental Information 1 SEM image of 2,000 mg/L CuO-NPs

10.7717/peerj.10351/supp-2 Supplemental Information 2 Effects of CuO NPs on growth and nitrogen removal of strain Y-11 with and without Fe^2+^ addition A is nitrate removal; B is ammonium removal. Error bars represent the standard deviation for three replicates.

10.7717/peerj.10351/supp-3 Supplemental Information 3 Effect of Fe^2+^ addition on Cu^2+^ dissolution from CuO NPs A is a nitrate source; B is an ammonium source. Error bars represent the standard deviation for three replicates.

10.7717/peerj.10351/supp-4 Supplemental Information 4 Effects of *Cu*^2+^ on growth and nitrogen removal of strain Y-11 with and without Fe^2+^ addition A is a nitrate source; B is an ammonium source. Error bars represent the standard deviation for three replicates.

10.7717/peerj.10351/supp-5 Supplemental Information 5 Effect of Fe^2+^ on the hydrodynamic diameter of CuO-NPs

## Additional Information and Declarations

### Data Availability

The following information was supplied regarding data availability: The Supplementary Information and original measurement data are available in the Supplementary Files.
