# Supplementary material for: Exogenous Fe2+ alleviated the toxicity of CuO nanoparticles on Pseudomonas tolaasii Y-11 under different nitrogen sources
Source: PeerJ. 2020 Nov 10;8:e10351. doi: 10.7717/peerj.10351 (PMC7664463; doi:10.7717/peerj.10351)
Supplement: Supplemental Information 1 [file peerj-08-10351-s001.docx]

Exogenous Fe^2+^ alleviated the toxicity of CuO nanoparticles on *Pseudomonas tolaasii* Y-11 under different nitrogen sources

Yuran Yang^1^, Can Zhang^1^, Xuejiao Huang^1^, Xuwei Gui^1^, Yifang Luo^2^, Zhenlun Li^1*^

^1^ Chongqing Key Laboratory of Soil Multiscale Interfacial Process, College of Resources and Environments, Southwest University, Chongqing, China

^2^ Chongqing Key Laboratory of plant disease biology, College of Plant Protection, Southwest University, Chongqing, China

^*^Corresponding Author:

Zhenlun Li

No. 2 Tiansheng Road, Tiansheng Street, Beibei District, Chongqing, 400715, China.

Email address: lizhlun4740@sina.com


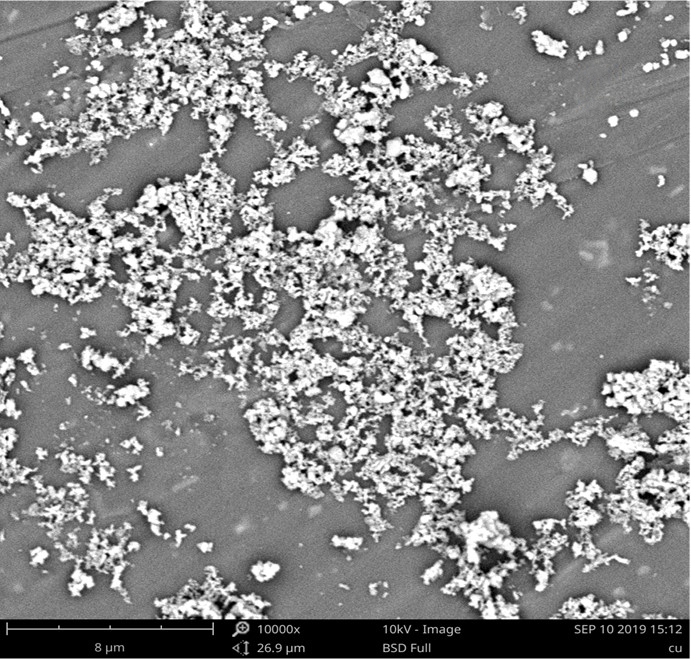


Suppl. Fig S1: SEM image of 2000 mg/L CuO-NPs.
